# Lack of Association Between *MIR155* Gene Variant rs767649 and Risk for Parkinson’s Disease

**DOI:** 10.3390/ijms27156943

**Published:** 2026-08-02

**Authors:** Hortensia Alonso-Navarro, Sofía Ladera-Navarro, Pedro Ayuso, Pau Pastor, Ignacio Álvarez, Miquel Aguilar, Elena García-Martín, José A. G. Agúndez, Félix Javier Jiménez-Jiménez

**Affiliations:** 1 Section of Neurology, Hospital Universitario del Sureste, 28500 Arganda del Rey, Spain; fjavier.jimenez@salud.madrid.org; 2 University Institute of Molecular Pathology Biomarkers, Universidad de Extremadura, 10071 Cáceres, Spain; soladeran@unex.es (S.L.-N.); payupar@unex.es (P.A.); elenag@unex.es (E.G.-M.); jagundez@unex.es (J.A.G.A.); 3 Unit of Neurodegenerative Diseases, Department of Neurology, University Hospital Germans Trias i Pujol, 08916 Badalona, Spain; pastorpau@gmail.com; 4 Department of Neuroscience, Germans Trias i Pujol Research Institute (IGTP), 08916 Badalona, Spain; ignacioalvafer@gmail.com; 5 Àptima Centre Clínic Sant Cugat del Vallès, 08174 Sant Cugat del Vallès, Spain; miquelaguilar@gmail.com

**Keywords:** *MIR155* gene, genetics, Parkinson’s disease, polymorphisms, risk-factors

## Abstract

An important role of neuroinflammation in the pathogenesis of Parkinson’s disease (PD) has been proposed. Since some microRNAs (miRNAs), and miR-155 in particular, are involved in neuroinflammatory processes, a previous study reported an association between one of the most common single nucleotide variants (SNVs), rs767649, in the *MIR155* gene, and risk for PD. The aim of the current study was to replicate this finding in a larger Spanish population case–control series. We analyzed genotype and allele frequencies of the *MIR155* rs767649 SNV in a Spanish Caucasian cohort consisting of 459 PD patients and 460 age- and sex-matched healthy controls, using a TaqMan allelic discrimination assay specifically designed for rs767649. The genotype frequencies of the *MIR155* rs767649, under codominant, dominant, recessive, and overdominant inheritance models, as well as allelic frequencies, showed no significant differences between PD patients and healthy controls. Likewise, the age at PD onset did not differ among the three *MIR-155* rs767649 genotypes. These data did not identify a statistically significant association between *MIR155* rs767649 variants and PD risk. However, the low frequency of the variant allele resulted in limited statistical power, and modest genetic effects cannot be excluded.

## 1. Introduction

Although Parkinson’s disease (PD) was described in 1817, more than 200 years ago, its etiopathogenesis has not yet been clearly established. Over the past 40 years, numerous studies have suggested that it is a complex disease from a genetic perspective, resulting from the interplay between various genetic and environmental factors, as well as aging. To date, according to the NCBI Gene Database, twenty-six genes associated with familial PD have been reported and named PARK1–PARK26; however, they account for only a small percentage of the heritability of PD (https://www.ncbi.nlm.nih.gov/gene, accessed on 8 April 2026). The results of both hypothesis-free genome-wide association studies (GWAS) and meta-analyses of hypothesis-driven case–control association studies on candidate genes (many of which have yielded inconsistent results) have provided some data on the possible role of certain genetic variants in the risk of developing PD [1]; however, a detailed discussion of these findings is beyond the scope of this article.

The proposed pathogenic mechanisms for PD include, among others, oxidative stress, endoplasmic reticulum stress, mitochondrial dysfunction, metabolic dysregulation, excitotoxicity, deficits in trophic factors, disrupted calcium homeostasis, and neuroinflammation, leading to programmed cell death (including apoptosis, ferroptosis, necroptosis, pyroptosis) and non-programmed cell necrosis [2,3]. Recent data have suggested a significant association between neuroinflammation and PD [2,4,5].

MicroRNAs (miRNAs) are a class of endogenous non-coding RNAs of approximately 21–25 nucleotides that occur naturally and are involved in the regulation of gene expression. Cancer, immune dysfunction, and some neurological diseases are associated with aberrant expression of these molecules. Specifically, the multifunctional miRNA miR-155 is implicated in processes such as innate [6,7] and adaptive immunity [8,9], hematopoiesis [7], cell proliferation and apoptosis [10], host defense [9], and inflammation [11,12,13,14,15]. miR-155 is upregulated by cytokines and pathogens and modulates the production of tumour necrosis factor alpha (TNF-α), interleukin-6 (IL-6), interleukin-1β (IL-1β), interferon-γ (IFN-γ), and interferon regulatory factor 3 (IRF3) [13,14].

miR-155 is encoded by the *microRNA-155* gene (*MIR155*, also named *MIRN155*, *miRNA 155* or *mir-155*; chromosome 21q21.3; gene ID 406947; MIM 609337; https://www.ncbi.nlm.nih.gov/gene/406947, accessed on 8 April 2026). One of the most common variants in the *MIR155* gene, the rs767649 single nucleotide polymorphism (SNP), is a T > A polymorphism that locates at the regulatory/promoter region of the gene and can affect miR-155 expression levels (with the TT genotype often associated with higher miR-155 expression levels) [16].

The measurement of blood levels of miR-155 has been the subject of several studies in patients with PD. Two studies conducted using peripheral blood mononuclear cells (PBMCs) from PD patients and controls showed contradictory results: one found upregulation [17], while the other showed downregulation of miR-155 in PD patients compared with controls [18]. Two studies reported upregulation of serum miR-155 levels in PD patients [19,20], one of which also identified a correlation between this parameter and PD severity, as well as a negative correlation on one cognitive scale [20]. Serum miR-155 levels have been found to be similar in males and females with PD [21,22] and remained unchanged in a two-year longitudinal study of PD patients [22]. Serum extracellular vesicle levels of miR-155 were reported to be associated with bradykinesia and tremor in PD patients [23]. In addition, an experimental study in rodents demonstrated upregulation of miR-155 in an in vivo model of PD produced by adeno-associated virus–mediated expression of α-synuclein (the aggregation of which into Lewy bodies is a defining pathological hallmark of PD), as well as a reduction in proinflammatory responses to this protein, which blocks neurodegeneration induced by α-synuclein in mice with a complete deletion of *Mir155* gene [24].

A recent case–control association study conducted in the Chinese Han population, involving 110 patients with PD and 120 healthy controls, showed a decreased risk of PD in subjects carrying the *MIR155* rs767649 A allele, as well as greater disease severity, poorer scores on a recognized cognitive scale, and higher serum levels of miR-155 in PD patients carrying the *MIR155* rs767649 TT genotype [20]. The present study aimed to replicate the potential role of rs767649 variants in PD risk in a larger cohort of Spanish Caucasian patients with PD and healthy controls.

## 2. Results

The *MIR155* rs767649 allelic frequencies were in Hardy–Weinberg equilibrium in both study groups, with *p*-values of 0.406 and 0.996, respectively, in PD patients and controls. The results of genotypes in PD patients and control subjects, according to codominant, dominant, recessive, and additive models, are summarized in Table 1, and those of allelic variants in Table 2. Under the codominant model, odds ratios were calculated separately for the comparisons AT versus TT and AA versus TT, using TT as the reference genotype. The extremely small number of AA homozygotes prevented a reliable evaluation of recessive effects.

No significant differences were found under any of the four genetic models in the frequency of the different genotypes between PD patients and healthy controls, either in the overall sample or after stratification by sex (Table 1). Similarly, the allele frequencies of the two variants did not differ significantly between the two groups, either in the overall analysis or after stratification by sex (Table 2). Finally, the age at PD onset and the evolution time of PD did not differ significantly among the three possible genotypes of the *MIR155* rs767649 variant (Table 3). To address potential residual confounding, a multivariate logistic regression analysis adjusted for age and sex was conducted. The adjusted results were consistent with those obtained in the univariate analyses and did not reveal any statistically significant association between rs767649 and Parkinson’s disease susceptibility.

## 3. Discussion

Since neuroinflammation seems to play an important role in the pathogenic mechanisms of PD [2,3,5], and miRNAs, especially miR-155, play an important role in neuroinflammation, it was reasonable to investigate the different variants of the *MIR* genes with the risk of developing PD in our population.

Most case–control association studies published on the possible association of *MIR* gene polymorphisms or variants with the risk of PD have failed to find such an association. Specifically, no association has been found with PD risk and the variants *MIR133b*-90delA [25], *MIR196a2* rs11614913 [26], *MIR4519 rs897984* [27], *MIR548at-5p* rs11651671 [27], *MIR126* rs4636297 [28], and certain variants of the *MIR433* gene [29]. A previous study showed an association between *MIR4697* rs329648 and the risk of PD [30], but this was not further replicated [31]. A recent study in the Chinese Han population showed an upregulation of miR-155 levels in PD patients, and a lower risk of developing PD in subjects carrying the *MIR155* rs767649A allele [20].

The molecular mechanisms of miR-155 in PD are not well understood, although several studies suggest a possible role for miR-155 in the pathogenesis of this disease:

(a) A study using a NanoString nCounter microRNA assay showed downregulation of miR-155 in the putamen of PD patients [32]. However, another study analyzing 733 miRNAs in the postmortem substantia nigra of eight PD patients and four controls found widespread alterations in miRNA expression, although miR-155 was not among the significantly differentially expressed miRNAs reported [33].
(b) In mice with parkinsonism induced by 1-methyl-4-phenyl-1,2,3,6-tetrahydropyridine (MPTP), miR-155-5p expression was found to be upregulated, promoting microglial activation, inflammation, apoptosis, and oxidative stress [34]. In addition, as previously mentioned, miR-155 expression has been reported to be upregulated in α-synuclein-based animal models of PD and in patients with PD [24].
(c) miR-155 is able to enhance inflammatory signaling pathways by promoting a pro-inflammatory microglial phenotype and suppressing anti-inflammatory regulators such as Suppressor of Cytokine Signaling 1 (SOCS1). This leads to increased production of pro-inflammatory cytokines, contributing to neuronal injury and neurodegenerative processes in the substantia nigra of patients with PD [35,36,37].
(d) Mutations in the *DJ-1* (*PARK7*) gene, which are associated with familial PD, cause a loss of DJ-1 function, leading to increased miR-155 expression, reduced *SOCS1* mRNA stability, and enhanced inflammatory responses in microglia and astrocytes [36].
(e) miR-155 is able to enhance Nuclear Factor Kappa B (NF-κB) signaling through inhibition of the negative regulator of inflammatory signaling, Src homology 2-containing inositol phosphatase 1 (SHIP-1). This leads to increased transcription of inflammatory genes, promoting microglial activation and dopaminergic neuron loss [38].
(f) miR-155 enhances the expression of enzymes such as inducible nitric oxide synthase (iNOS) and cyclooxygenase-2 (COX-2), leading to increased nitric oxide production, oxidative stress, and mitochondrial dysfunction, which contribute to dopaminergic neuronal loss [35].

To place our findings in the context of currently available large-scale genetic data, we searched the NHGRI-EBI GWAS Catalog, the Open Targets Genetics platform, and the GWAS Atlas for associations involving rs767649 and Parkinson’s disease. No genome-wide significant association between this variant and Parkinson’s disease was identified in these resources at the time of consultation. The lack of evidence from these large genetic resources is consistent with our findings and does not support a major role of rs767649 in PD susceptibility. Nevertheless, given the low minor allele frequency of this variant and the limited statistical power of both candidate-gene and GWAS approaches to detect very small effects, a modest contribution to PD risk cannot be completely excluded.

In the current study, we failed to replicate the association of the *MIR155* gene variant rs767649 with PD reported in a previous study [20], using a larger sample size. We found no association between this variant and PD risk, either in the overall sample or after stratification by sex. We also found no influence of the *MIR155* rs767649 variant on the age at PD onset. The discrepancy between the results obtained in the Chinese Han population and those observed in our Spanish Caucasian cohort may be related to population-specific genetic factors. Parkinson’s disease is a complex disorder in which the effect of individual genetic variants may depend on interactions with other susceptibility loci whose frequencies differ among populations. In addition, rs767649 itself may not be the causal variant but rather a marker in linkage disequilibrium with a nearby functional variant. Differences in linkage disequilibrium patterns between ethnic groups could therefore lead to the detection of an association in one population but not in another. These possibilities should be considered when interpreting the inconsistent findings across studies and warrant further investigation in larger cohorts from diverse ancestral backgrounds.

Several limitations of our study should be acknowledged. First, patient recruitment was conducted in a hospital setting, and therefore some degree of selection bias cannot be completely excluded. In addition, both the PD patients and control subjects included in the present study had previously participated in genetic association studies conducted by our research group. Although these participants were recruited independently of the current investigation and according to predefined inclusion criteria, this circumstance may further limit the generalizability of the findings to other populations. Second, no comparable studies evaluating rs767649 in European Caucasian PD populations are currently available, limiting direct comparisons with our findings. Third, we did not perform functional analyses evaluating miR-155 expression according to rs767649 genotype. Although such analyses could have provided valuable biological support for the genetic findings, suitable samples were not available for all participants, and the extremely small number of AA homozygotes in our cohort would have limited the interpretability of genotype-expression comparisons. Future studies integrating genotyping with measurements of miR-155 expression in serum, plasma, or PBMCs may help clarify the functional significance of this variant.

Finally, the low frequency of the rs767649 A allele, particularly the very small number of AA homozygotes (one control and two PD patients), resulted in wide confidence intervals and limited statistical power, reducing the precision of the estimated odds ratios and limiting our ability to detect modest genetic effects. Consequently, although no statistically significant association between rs767649 and PD risk was identified, a type II error cannot be excluded, and our findings should be interpreted as showing no evidence of association rather than definitively excluding a contribution of this variant to PD susceptibility. Substantially larger cohorts, as well as independent replication studies in other populations, will be required to more precisely evaluate the potential role of rs767649 in Parkinson’s disease and to adequately assess the small effect sizes that may be associated with this variant.

On the other hand, although the possibility that some of the healthy controls who participated in the study may develop PD in the future cannot be ruled out, taking into account the PD incidence rates described in Spain for individuals over 65 years old [39], as well as the proportion of subjects in the healthy control group who carried the different genotypes of the variant analyzed, it is very unlikely that this factor would have had a significant influence on the results.

In conclusion, after considering all the limitations of the study, our results do not support a significant association between the *MIR155* rs767649 variant and PD risk or age at disease onset in this Spanish Caucasian cohort. However, given the low statistical power of the study, these results are inconclusive rather than definitively negative; therefore, a modest genetic effect cannot be excluded, and larger studies in independent populations are warranted to further clarify the possible role of this variant in PD susceptibility.

## 4. Materials and Methods

### 4.1. Study Participants

In this study, 459 patients diagnosed with PD participated according to the UK Parkinson’s Disease Society Brain Bank Clinical Diagnostic Criteria [40], along with 460 healthy controls matched by age and sex. Both PD patients and controls were over 18 years of age.

PD patients were recruited from the Movement Disorders Units of four hospitals and were examined before inclusion by clinical neurologists with extensive experience in the diagnosis of Parkinson’s disease. Controls were recruited from Hospital Universitario Infanta Cristina in Badajoz, Spain (most of them staff or students of the hospital) and from Clínica Universitaria de Navarra in Pamplona, Spain (spouses of PD patients).

None of the healthy controls included in the study had a family history of PD, other movement disorders, or other neurological diseases. Recruitment of both PD patients and controls was carried out between 2003 and 2018.

The demographic data of both PD patients and control subjects, who have participated in previous case–control association studies [41,42], are summarized in Table 4. All participants were self-reported Spanish Caucasians of European ancestry. Because detailed ancestry-informative markers were not available, formal ancestry-stratified analyses could not be performed. We agree that recruitment of some controls among spouses of PD patients may represent a potential source of selection bias, and we have acknowledged this limitation in Section 3.

### 4.2. Ethical Aspects

The study was approved by the Ethics and Clinical Research Committees of the Clínica Universidad de Navarra (Pamplona, Spain; approval code 123/2013; 19 February 2013), Hospital Universitari Mutua de Terrassa (Terrassa, Barcelona, Spain; approval code EO/1882; 28 November 2018), and Hospital Universitario Infanta Cristina (Badajoz, Spain; no approval code; 1 April 2004). The study complied with the principles of the Declaration of Helsinki in force prior to the beginning of recruitment, and written informed consent from all participants was a mandatory requirement for inclusion.

### 4.3. Genotyping of MIR155 rs767649 Variant

Genotyping *of MIR155* rs767649 was performed by using real-time PCR (Applied Biosystems 7500 qPCR thermocycler, Waltham, MA, USA) with specific custom-designed TaqMan assay (C___2212229_10, Life Technologies, Alcobendas, Madrid, Spain) on genomic DNA obtained from peripheral leukocytes of venous blood samples of patients diagnosed with PD and controls.

### 4.4. Statistical Analysis

The Hardy–Weinberg equilibrium in both PD patients and controls was assessed using the online application (https://codingace.net/biology/hardy_weinberg.html, accessed on 8 April 2026) and the statistical package PLINK version 1.9, release beta 7.11 [43] was used to analyze allele and genotype frequencies. The remaining statistical analyses were carried out using SPSS version 20.0 (SPSS Inc., Chicago, IL, USA). Intergroup comparisons were performed using the χ^2^ test or Fisher’s exact test, as appropriate, and correction for multiple testing was performed using the false discovery rate procedure [44]. For each intergroup comparison, including all genotypes or all alleles, both crude and corrected values (*p* and *Pc*, respectively) were calculated.

The sample size was calculated according to the minor allele frequency in control subjects for a relative risk (RR) value of 1.5 (*p* < 0.05) [45,46]. However, because the observed minor allele frequency was approximately 5%, the effective power to detect small genetic effects was limited, particularly for analyses involving homozygous carriers of the variant allele. The statistical power for the variant allele in the current study, based on the allele frequencies observed in healthy controls and patients for one-tailed association, was 7.4.%, and for two-tailed associations was 3.94%. Negative predictive values were also calculated [47]. The comparison of age at PD onset across genotype categories for the SNP studied was performed using a *t*-test for independent samples.

The reporting of the study was conducted in accordance with the EQUATOR Network guidelines [48]. In this regard, Table S1 summarizes the STROBE and STREGA checklists.

## Figures and Tables

**Table 1:** Genotypes of patients with PD and healthy volunteers (control subjects) according to different genetic models. TT was used as the reference genotype. Under the codominant model, two ORs are reported corresponding to AT vs. TT and AA vs. TT comparisons. OR: odds ratio; CI: confidence interval; *p*: crude *p* value; *Pc*: adjusted *p* value.

Variant		Genotype, n (%)		Codominant OR (95% CI): AT vs. TT; AA vs. TT	*p*, *Pc*	Dominant OR (95% CI)	*p*, *Pc*	Recessive OR (95% CI)	*p*, *Pc*	Overdominant OR (95% CI)	*p*, *Pc*
**Whole series**											
**rs767649**	**TT**	**AT**	**AA**								
Control	418 (90.87%)	41 (8.91%)	1 (0.22%)								
PD	415 (90.41%)	42 (9.15%)	2 (0.44%)	1.03 (0.66–1.62) 2.01 (0.18–22.30)	0.790, 0.910	1.06 (0.68–1.65)	0.820, 0.910	2.01 (0.18–22.23)	0.620, 0.910	1.03 (0.66–1.62)	0.910, 0.910
**Women**											
**rs767649**	**TT**	**AT**	**AA**								
Control	228 (93.4%	15 (6.1%)	1 (0.41%)								
PD	217 (88.9%)	26 (10.7%)	1 (0.41%)	1.82 (0.94–3.53) 1.05 (0.07–16.9)	0.200, 0.267	1.77 (0.93–3.38)	0.109, 0.218	1.00 (0.06–16.08)	1.000, 1.000	1.82 (0.94–3.53)	0.102, 0.218
**Men**											
**rs767649**	**TT**	**AT**	**AA**								
Control	190 (88.0%)	26 (12.0%)	0								
PD	198 (92.1%)	16 (7.4%)	1 (0.5%)	0.59 (0.31–1.14) 2.88 (0.12–71.12)	0.278, 0.371	0.63 (0.33–1.19)	0.198, 0.371	3.03 (0.12–74.75)	0.499, 0.499	0.59 (0.31–1.13)	0.143, 0.371

**Table 2:** Allele frequencies in patients with PD and healthy volunteers (control subjects). OR: odds ratio; CI: confidence interval; *p*: crude *p* value; *Pc*: adjusted *p* value; NPV: negative predictive value.

Variant	Allele, n (%)		OR (95% CI)	*p*, *Pc*	NPV (95% CI)
**Whole series**					
**rs767649**	**T**	**A**			
Control	877 (95.3%)	43 (4.7%)			
PD	872 (95.0%)	46 (5.0%)	1.076 (0.70–1.65)	0.737	0.50 (0.50–0.51)
**Women**					
**rs767649**	**T**	**A**			
Control	471 (96.5%)	17 (3.5%)			
PD	460 (94.3%)	28 (5.7%)	1.08 (0.55–2.13)	0.815, 0.815	0.04 (0.03–0.05)
**Men**					
**rs767649**	**T**	**A**			
Control	406 (94.0%)	26 (6.0%)			
PD	412 (95.8%	18 (4.2%)	0.68 (0.37–1.26)	0.222, 0.444	0.04 (0.03–0.06)

**Table 3:** Age at onset and evolution time of PD according to the genotypes.

	Age at Onset (SD) Years	Two-Tailed *t*-Test *p* Value (TT vs. AT)	Two-Tailed *t*-Test *p* Value (TT vs. AA)
**rs767649 TT**	57.37 (12.68)		
**rs767649 AT**	58.14 (10.30)	0.704	
**rs767649 AA**	44.00 (5.66)	0.138	0.063
	**PD evolution time (SD) years**	**Two-Tailed *t*-Test *p* value (TT vs. AT)**	**Two-Tailed *t*-Test *p* value (TT vs. AA)**
**rs767649 TT**	9.23 (7.20)		
**rs767649 AT**	9.74 (6.79)	0.675	
**rs767649 AA**	10.33 (3.06)	0.882	0.791

**Table 4:** Demographic data.

Group	PD Patients (n = 459)	Controls (n = 460)	*p*
Age, y, mean (SD)	66.38 (11.87)	65.56 (10.54)	0.936
Age range, y	22–95	27–92	–
AAO, y, mean (SD)	57.39 (29.88)	NA	–
AAO range, y	14–85	NA	–
PD evolution time, y, mean (SD)	9.36 (7.17)	NA	–
PD evolution time, range, y	0–51	NA	–
Men n (%)	215 (46.8)	216 (47.0)	0.928

PD = Parkinson’s disease; y = years; AAO = age at onset; SD = standard deviation; NA = not available.

## Data Availability

All data relating to the current study, intended for reasonable use, are available from J.A.G. Agúndez (University Institute of Molecular Pathology Biomarkers, University of Extremadura -UNEx ARADyAL Instituto de Salud Carlos III, Av/de la Universidad S/N, E10071 Cáceres, Spain) and Hortensia Alonso-Navarro and F.J. Jiménez-Jiménez (Section of Neurology, Hospital del Sureste, Arganda del Rey, Madrid, Spain).

## Supplementary Materials

The following supporting information can be downloaded at https://www.mdpi.com/article/10.3390/ijms27156943/s1.
